# A Hypothesis of the Interaction of the Nitrergic and Serotonergic Systems in Aggressive Behavior Induced by Exposure to Lead

**DOI:** 10.3389/fnbeh.2018.00202

**Published:** 2018-09-03

**Authors:** Juan Carlos Martínez-Lazcano, Alfredo López-Quiroz, Rocío Alcantar-Almaraz, Sergio Montes, Alicia Sánchez-Mendoza, Mireya Alcaraz-Zubeldia, Luis Antonio Tristán-López, Beatriz Eugenia Sánchez-Hernández, Adriana Morales-Martínez, Camilo Ríos, Francisca Pérez-Severiano

**Affiliations:** ^1^Departamento de Neuroquímica, Instituto Nacional de Neurología y Neurocirugía (INNN), Mexico City, Mexico; ^2^Departamento de Neurofisiología, Instituto Nacional de Neurología y Neurocirugía (INNN), Mexico City, Mexico; ^3^Departamento de Farmacología, Instituto Nacional de Cardiología Ignacio Chávez, Mexico City, Mexico; ^4^Departamento de Genética, Instituto Nacional de Ciencias Médicas y Nutrición Salvador Zubirán, Mexico City, Mexico

**Keywords:** lead exposure, central nervous system, epigenetic, nitrergic and serotonergic systems, aggressive behavior

## Abstract

The effects caused by exposure to lead (Pb) are still considered as a relevant health risk despite public policies aimed to restricting the use of this element. The toxicity limit in the blood (10 μg/dL, established by the Center for Disease Control and Prevention) has been insufficient to prevent adverse effects and even lower values have been related to neurobehavioral dysfunctions in children. Currently, there is not a safe limit of exposure to Pb. A large body of evidence points to environmental pollutant exposure as the cause of predisposition to violent behavior, among others. Considering the evidence by our group and others, we propose that Pb exposure induces alterations in the brain vasculature, specifically in nitric oxide synthases (NOS), affecting in turn the serotonergic system and leading to heightened aggressive behavior in the exposed individuals. This review article describes the consequences of Pb exposure on the nitrergic and serotonergic systems as well as its relationship with aggressive behavior. In addition, it summarizes the available therapy to prevent damage in gestation and among infants.

## Introduction

### Lead and Its Effects as Public Health Matter

Lead (Pb) is a non-essential element for humans. It is considered one of the most common toxic metals in the environment and is frequently used in a variety of products, primarily in lead car batteries. Other uses of lead include leaded gasoline, paints, ceramics, ammunition, water pipes, solders, cosmetics, hair dye, farm equipment, airplanes, shielding for x-ray machines and in the manufacture of corrosion and acid resistant materials used in the building industry (Sanders et al., 2009). The chronic exposure to this pollutant remains a public health problem that affects several human body systems including nervous, reproductive, and circulatory. In bones, nails and teeth, Pb is deposited and may remain accumulated throughout life. In pregnant women, it has been reported that Pb can cross the placental barrier and is equally distributed in the mother and fetus (Fontana et al., 2013). Children are the most susceptible population to Pb exposure: they have been reported to show alterations in cardiac function, encephalopathy, genotoxicity, cognitive deficits, language alterations, antisocial behavior and juvenile delinquency after exposure to the metal (Amos-Kroohs et al., 2016; Sampson and Winter, 2018). Evidence suggests that the rate of violent crimes in several countries is related to the levels of Pb in blood among the population, even in individuals at school age. The harmful effects of Pb have been described to be present up to 20 years after the initial exposure, evidencing that the metal causes short- and long-term effects (Carpenter and Nevin, 2010). Lead enters the human body when we consume contaminated water, breathe a polluted environment and use contaminated pottery still releasing Pb in the kitchen or when storing water, for example. Recent cases as the water poisoning in Flint, Michigan in 2015 and the evacuation of an entire neighborhood contaminated by a smelting plant in East Chicago, Indiana in 2016 have shed light on the contemporary perils of lead contamination (Goodnough, 2016a,b). Although environmental reforms such as the bans on Pb in gasoline and paint in the 1970’s were deemed victories for public health (Needleman, 2004; Markowitz and Rosner, 2013), Pb toxicity is far from being a hazard of the past. High levels of the metal have recently been found in several cities in the United States (Pell and Schneyer, 2016) and in both developed and developing countries around the world (Tong et al., 2000). The situation is especially important to Mexico because of the prevailing high environmental pollution and exposure to Pb in lead-glazed pottery that does not comply with the official standards. The Mexican government has passed bills related to the production and evaluation of ceramic (Norma Oficial Mexicana, 2007) as well as a program encouraging ceramic producers to use alternative unleaded glaze in pottery (National Program to Adopt Lead-Free Glazed Pottery, FONART, Mexico). Despite these actions, the levels of Pb in blood among the population are a concern mainly because of the levels of Pb in the environment, (Fontana et al., 2013; Amos-Kroohs et al., 2016), the easy access of the metal to the body and the manufacturers’ non-compliance with official standards (Norma Oficial Mexicana, 2007; Diaz-Ruiz et al., 2017). Therefore, Pb remains an environmental risk in countries like Mexico regardless of the restrictions in its use.

## How to Address the Problem of Neurological Alterations Due to Pb Exposure and Their Relationship With Behavioral Changes?

It is widely recognized that Pb exposure exerts toxic effects in every organ system, especially the central nervous system (CNS). Brain damage induced by Pb may result in a variety of neurological disorders, including mental retardation (Sanders et al., 2009) Alzheimer’s disease, Parkinson’s disease (Monnet-Tschudi et al., 2006) and schizophrenia (Opler et al., 2008). In addition, the neurotoxic effects of lead exposure may cause behavioral problems such as attention deficit hyperactivity disorder, juvenile delinquency, and criminality (Pihl and Ervin, 1990; Wright et al., 2008). In addition, lead can adversely affect general intellectual functioning (Sanders et al., 2009), visuospatial function (Weisskopf et al., 2007), and verbal learning and memory (Bleecker et al., 2005). Furthermore, recent studies have demonstrated that executive function may be at risk in Pb-induced neurotoxicity (Trope et al., 2001; Canfield et al., 2004). Most works on executive dysfunction focus on children and only a few aim to analyze adult workers exposed to Pb (Trope et al., 2001; Canfield et al., 2004; Sanders et al., 2009). Since adult occupational exposure and childhood developmental exposure to Pb cause cognitive and behavioral alterations (Sanders et al., 2009; National Toxicology Program, 2012), studies on workers exposed to the pollutant is needed to clarify Pb neurotoxicity related to executive functions.

The legacy of Pb, both historical and contemporary, carries important theoretical implications for the study of crime. An association between lead exposure and cognitive impairment has been documented in medical research (Lanphear et al., 2000; Canfield et al., 2003; Reuben et al., 2017), and several criminologists have long argued that there is a connection between cognitive ability and delinquency (Farrington, 1998). Similarly, there is evidence that Pb aggravates hyperactivity, impulsive behavior, and mental health problems (Braun et al., 2006; Winter and Sampson, 2017), which have been shown to predict delinquency (Elliott et al., 1989; Moffitt, 1990; Wright et al., 2008). Reyes (2015) reported an association between Pb exposure and subsequent delinquency and crime activities.

Animal models must be used to evaluate the molecular-biological processes related to exposure to pollutants. Particularly, Pb is orally administered to the pregnant and/or lactating female to evaluate the resulting consequences of chronic exposure to low levels of Pb in young animals during gestation and development. The experimental models that start with exposure during gestation and conclude at weaning cover most of the neurogenesis and neuronal development (Virgolini and Cancela, 2014). In spite of its limitations, the oral administration of Pb to the pregnant mother and/or infant is the animal model most frequently used to evaluate the consequences of chronic exposure to low levels of Pb during development. The model allows for behavioral evaluation of the subjects after 70–90 days of exposure and age (de Souza Lisboa et al., 2005; Weston et al., 2014). Additionally, this scheme of exposure enables the evaluation of epigenetic effects (DNA methylation, histone modifications, and ncRNAs regulation) induced by exposure to Pb. This administration scheme has allowed researchers to evaluate the effect of prenatal exposure to lead and the predisposition to aggressive behavior as well as the correlation between these events and the regulation of the nitrergic and serotonergic systems in the brain. The notions of a relationship between serotonergic and nitrergic systems and between these systems and aggressiveness have been recently explored by studies that evaluated the effect of the inhibition of neuronal nitric oxide synthases (nNOS) activity on the serotonergic system and its relationship with aggressive behavior (Carreño-Gutiérrez et al., 2017). This type of studies helps to identify the molecular targets of the damage produced by exposure to Pb, the degree of damage in brain development and the behavioral alterations caused.

## Lead as a Possible Cause of Aggressive Behavior

The exposure to environmental pollutants as a predisposition factor of violent behavior has received little attention since violent and antisocial behaviors are usually attributed to socioeconomic factors, including poverty, deficient education and family instability. There is evidence that violent behavior is more frequent among individuals with low intellectual quotient (Amos-Kroohs et al., 2016), a neurobehavioral pattern that has been described in children exposed to Pb at an early stage. Recently, Sampson and Winter (2018) published an article about the social consequences of exposure to lead, highlighting criminal behavior in young people exposed to lead, and suggesting that some factors can be explained through the hypothesis of displacement of Ca^++^ by Pb^++^ (Sampson and Winter, 2018). The toxicity mechanisms through which Pb promotes neurological alterations, in particular aggressiveness, in the CNS have not been fully described in humans or experimental models. In this review article, we propose a molecular hypothesis that involves modifications in the activity and expression of nNOS, and a consequent modification in serotonin 5-hydroxytryptamine (5-HT) and its 5-HT1B receptor levels in animals that present an increase in aggressive behavior after lead exposure. It is important to note that there are other neurotransmitters and hormonal systems involved in aggressive behavior, such as dopamine and testosterone. Additionally, there is evidence indicating that NOS are Pb targets in the brain vasculature (García et al., 1999). Moreover, some reports indicate that the decrease in nitric oxide (NO) has an effect upon the serotonergic pathway and aggressive behavior (Chiavegatto and Nelson, 2003; Martínez-Lazcano et al., 2007; Sansar et al., 2012). In Soria et al. (2008) collected and integrated available information on the participation of 5-HT in the modulation of aggressive behavior in several animal species, including humans.

Considered an inhibitor of aggressiveness, 5-HT reduces impulsivity at high concentrations while its reduction leads to increased intensity and frequency of aggressive and antisocial reactions that are more impulsive than premeditated. The exact mechanisms of Pb neurotoxicity and its effects on the aminergic system in rat brain remain unclear, but they might exist at different levels, altering the concentration of 5-HT and its metabolite, 5-hydroxyindoleacetic acid (5-HIAA), suggesting an effect of Pb on L-Monoamine oxidases (MAO), a family of enzymes that catalyze monoamine oxidation (Basha et al., 2012). There are reports on dissociation from synthesis, storage, and release of 5-HT with effects on the formation of 5-HIAA (Lasley et al., 1984) and alterations in both the synthesis and metabolism of serotonin receptors (Leret et al., 2002; Sansar et al., 2012). In consequence, Pb produces an alteration in brain functions, which could be attributed to an imbalance in endogenous neurotransmitters, like dopamine and 5-HT. Because of this, human and animal exposure to Pb during early development might alter the normal levels of 5-HT, metabolite 5-HIAA and the serotonin transporter (5HTT; Hou et al., 2012), producing significant changes in 5-HT replacement and, in consequence, aggressive behavior (Wang et al., 2012).

## Evidence of the Relation Between NOS, 5-HT and Aggressiveness

In mice and rats, male aggression is a resource to exclude other males from a breeding unit or maintain dominance within a more complex social structure. From an evolutive perspective, aggressive behavior displayed by the dominant male is highly adaptive since territorial males have access to breeding; therefore, they have greater reproductive success than subordinates and non-territorial males. In this case, there is a close relationship between the ability to fight, rank, and perform breeding events (Ferrari et al., 2006). In unstable rodent social groups, recurring conflicts compromise the immune system, divert energy and time of breeding and foraging, increase the risk of injury, interrupt circadian rhythms of endocrine and cardiovascular functions, and shorten lifespan (Fleshner et al., 1989; Stefanski, 2001). Studies carried out by Carreño-Gutiérrez et al. (2017) demonstrated the relationship between the nitrergic and serotonergic systems. Using zebra fish and knockout mice (−/–), they showed a decrease in the production of NO and described a reduction of 5-HT in MAO activity and dopamine levels. Also, the treatment of rats with NO donor molsidomine increased 5-HT metabolism (Lorenc-Koci et al., 2013) whereas nNOS inhibition with Nω-nitro-L-arginine (L-NAME) decreases neurotransmitter turnover in mice (Karolewicz et al., 2001), further linking NO to MAO. In 1999, our team showed that mice exposed to doses of lead acetate (250, 500 and 1,000 ppm) in drinking water showed dose-dependent increasing concentrations of Pb in brain tissue. These results correlated with the activity of calcium-dependent NOS (cNOS; García et al., 1999) and learning and memory (García et al., 2004). Endothelial NOS (eNOS) and nNOS, both calcium-dependent, participate in the control of vascular activity in the CNS. Then, the alterations found in cNOS activity might also affect vascular functions in the CNS. In some cases, aggressive behavior is attributed to cognitive deficits, speech alterations and low intellectual quotient. Several studies have demonstrated that NOS is involved in plasticity brain changes underlying long term potentiation (LTP) in the hippocampus (Hawkins et al., 1998; Ko and Kelly, 1999; Schuman and Madison, 1991) and formation of memory in several tasks (Böhme et al., 1993; Zhang et al., 1998; Zou et al., 1998). García et al. (2004) proved that the exposure to Pb blocks LTP induction in the hippocampus, which is associated to a decrease in the number of tasks involving learning and memory. Knock-out mice for eNOS and nNOS show reduction and loss of LTP (Hopper and Garthwaite, 2006) as well as increased aggressive behavior (Huang et al., 1993, 1995). Furthermore, eNOS knock-out mice show severe alterations in angiogenesis, leading to vascular problems.

**Figure 1:**
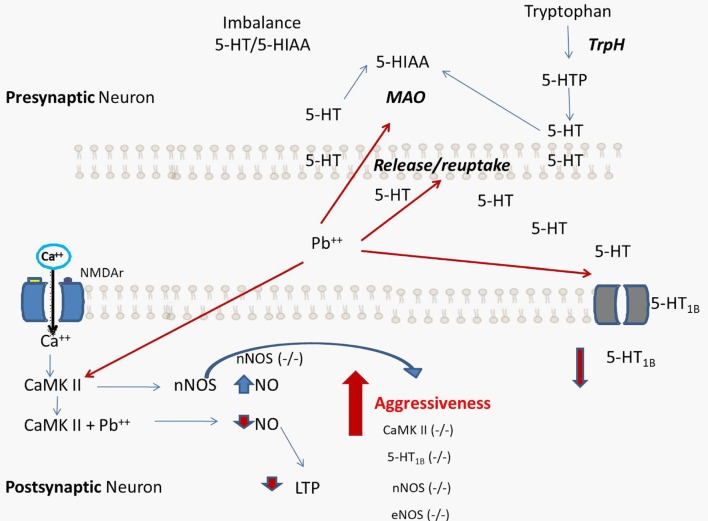
Proposed hypothetical scheme that summarizes the processes caused by lead (Pb) exposure. The increase in aggressive behavior is a direct consequence of alterations in the vascular-nitrergic-serotonergic system by interaction with Pb. The processes that lead to neurochemical alterations caused by Pb coincide with the behavioral alterations found in genetically modified experimental models, such as knock-out mouse for 5-HT_1B_ (−/−), endothelial NOS (eNOS; −/−), and neuronal nitric oxide synthases (nNOS; −/−). 5-HT: serotonin, 5-HIAA: 5-hydroxyindoleacetic acid, MAO: monoamino oxidase, TrpH: tryptophan Hydroxylase.

A significant portion of individual differences in aggressive behavior have been attributed to genetic factors. For this reason, many studies have conducted research on aggressive behaviors, in endogamic and exogamic mouse strains and recombinant or selected lines, to map or identify genes of aggressive behavior and determine the mechanisms through which genes influence aggression. These classic genetic studies have selected animals with different levels of aggressive behavior, suggesting that the tendency of getting involved in an intra-specific attack is a hereditary characteristic (Ferrari et al., 2006). In addition to these studies, the knock-out mouse generation produced strains that developed increased aggressive behavior, such as 5-HT1B (−/−), eNOS (−/−), and nNOS (−/−), among others. Furthermore, nNOS (−/−) mice show alterations in 5-HT metabolism (Chiavegatto and Nelson, 2003) while eNOS (−/−) animals display vascular alterations. Based on this information, increased aggressive behavior by Pb exposure is likely a direct consequence of alterations in the vascular-nitrergic-serotonergic systems (Figure 1). Additionally, NO has already been shown to modulate monoamine reuptake by indirect phosphorylation or direct S-nitrosylation of SERT, NET and DAT (Miller and Hoffman, 1994; Chanrion et al., 2007). Similarly, MAO activity is reduced by phosphorylation or S-nitrosylation. A negative feedback loop acting at gene transcription level could then lead to heightened levels of MAO expression in compensation for the reduced enzyme activity. Also, NO can alter neurotransmitter release via phosphorylation of synaptosomal proteins (Hirsch et al., 1993), thereby altering the amount of time in which neurotransmitters interact with their receptors.

## Preventive and Therapeutic Treatment Schemes to Prevent the Entry of Pb to the Organism

Maternal Pb exposure significantly influences embryonic and fetal development and is directly related to pregnancy impairment outcomes, including low birth weight, intellectual and learning disabilities and abnormal behavior (Sanders et al., 2009). Therefore, the design of studies on exposure to Pb during the embryonic stage aimed to create schemes or therapeutic treatments for preventing exposure to Pb and/or stopping any mechanism of damage related to this metal. For these reason, actions for detoxification and therapeutic chelation strategies are necessary. Some of the current therapeutic proposals are explained below.

### Ascorbic Acid

In a study by Chang et al. (2012), pregnant Sprague-Dawley rats exposed to 0.2% Pb were treated with ascorbic acid (100 mg/kg/day) during pregnancy and lactation. The study proved that the treatment could ameliorate Pb-induced oxidative damage in the developing hippocampus and recover protein levels of Cu/ZnSOD, MnSOD and catalase (CAT). Ascorbic acid also reduced the number of damaged cells in cornu ammonis 1 and 2 and dentate gyrus of hippocampus. Similarly, Massó-González and Antonio-García (2009) showed that ascorbic acid attenuates Pb-induced oxidative stress in liver and kidney using *in vitro* and *in vivo* models. However, the precise mechanism by which ascorbic acid is responsible for the beneficial effects on antioxidant systems in the developing rat brain remains unclear. These results might help to find protection strategies to prevent developmental deficits and cognitive dysfunctions observed in childhood after *in utero* low-level Pb exposure (Chang et al., 2012).

### Antioxidant MitoQ

MitoQ is a known mitochondrial-specific antioxidant with ONOO-scavenging activity that accumulates 500-fold in the mitochondrial matrix by the use of the mitochondrial membrane potential. This antioxidant efficiently mitigated mitochondrial complexes II, III and IV inhibition, ONOO-mediated inhibition, increased mitochondrial ATP production, restored mitochondrial membrane potential, and decreased caspases 3 and 9 activity upon Pb exposure. MitoQ also suppressed synaptosomal lipid peroxidation and protein oxidation, showing diminished nitrite production 3-nitrotyrosine protein binding. A treatment based on 500 μg MitoQ administered orally to rats for 15 days after exposure to 100–400 ppm lead acetate in drinking water protected against damage induced by the metal. Also, SHSY5Y cells exposed to increasing concentrations of Pb (5, 15 and 15 μM) showed a dose-dependent loss of cell viability, which was reverted by concomitant presence of MitoQ (50 nM). MitoQ has been also proved to reduce alterations by mitochondrial dysfunction in both *in vivo* and *in vitro* models of Pb exposure and ameliorate oxidative and nitrosative parameters, demonstrating to be an effective neuroprotective agent against Pb neurotoxicity (Maiti et al., 2017).

### Zinc Diet

Recently, Kumar et al. (2017) reported that zinc administered as a dietary supplement (10 and 20 mg/kg) promoted immune-biochemical plasticity and protected against multiple stresses in Pb exposure damage in fish (*Pangasius hypophthalmus*). Zinc is a key essential element that acts as a growth promoter and plays a significant part in several other cellular functions including cell proliferation, cofactor reproduction, immune function and defense against free radicals. It’s considered a vital intracellular trace element for genetic stability and function. Zinc acts as cofactor for several metabolic pathways in many enzymatic systems and is also a major component of a number of metalloenzymes, such as carbonic anhydrase, carboxypeptidase, alcohol dehydrogenase, glutamic dehydrogenase and superoxide dismutase (Kumar et al., 2017).

### β-Asarone (cis-2.4,5-trimethoxy-1-allyl phenyl)

β-asarone is an active oriental herb component which reverts damage inflicted by Pb over spatial memory, possibly through synaptogenesis (Yang et al., 2016). Developmental rat pups were exposed to Pb throughout lactation period and β-asarone (10, 40 mg/kg, respectively) was administered intraperitoneally from postnatal day 14–21. Also, adult rats were exposed to Pb from embryo stages to 11 weeks of age and β-asarone (2.5, 10, 40 mg/kg, respectively) was given rats aged 9–11 weeks. β-asarone crossed the blood brain barrier and attenuated Pb-induced spine density reduction in hippocampal CA1 and dentate gyrus areas in a dose-dependent manner both in developmental and adult rats. Additionally, it has been proved that Pb-induced impairments of learning and memory are partially reverted after treatment with this molecule. Also, β-asarone effectively up-regulates the protein expression of NR2B, Arc and Wnt7a, which have been suppressed by Pb exposure. The above mentioned results suggest neuroprotective properties of β-asarone against Pb-induced memory impairments and an effect possibly related to the regulation of synaptogenesis, mediated via Arc/Arg3.1 and Wnt pathway. Traditional use and clinical reports showed that β-asarone is effective for the treatment of learning and memory deficits; therefore, it is likely to manage memory impairment following chronic Pb exposure (Yang et al., 2016).

## Perspectives and Conclusions

The signs and symptoms induced by the Pb exposition can appear immediately after exposure or may be delayed and include loss of memory, vision, cognitive and behavioral problems, and brain damage/mental retardation. Most early studies concentrated on the neurocognitive effects of lead, but recently higher exposures have been associated with such morbidities as antisocial behavior, delinquency, and violence (Hwang, 2007). Several hypotheses have been proposed to explain the mechanism of lead toxicity on the CNS, including the participation of the nitrergic and serotonergic systems. After considering the information available, we propose that the increase of aggressive behavior is a consequence of alterations in the vascular-nitrergic-serotonergic system by interaction with Pb, without forgetting the influence that other molecules such as dopamine and testosterone could have on aggressive behavior. However, several experimental studies are still needed to fully reaffirm this hypothesis, so that a pharmacological strategy can be proposed as a therapeutic target and applied from the embryonic stage to restore the neurological damage caused by lead in the CNS. Studies will have to obtain the scientific proof to demonstrate that the exposure to Pb promotes violent behaviors among the subjects exposed to the metal. This behavioral alteration currently represents a serious matter of public health and carries an elevated social cost.

Additionally, we seek to prevent and/or reduce environmental pollution by Pb and other toxic particles to prevent irreversible brain damage in adolescence and development of CNS from gestation stages. This panorama demonstrates the lack of knowledge regarding the mechanism of neuronal damage caused by the exposure to a pollutant and, at the same time, the need to recreate human environmental exposure at experimental level to obtain further understanding of the damage mechanisms in brain development and altered behavior.

## Author Contributions

All authors had full access to all the information in this review article and take responsibility for the integrity. JM-L, FP-S, AL-Q and RA-A designed the review. SM, MA-Z, CR, BS-H and LT-L contributed to order the bibliographic work of lead exposition and epigenetic consequences. AS-M and JM-L designed the hypothetic scheme. FP-S, AM-M and JM-L wrote the manuscript and had primary responsibility for the final content.

## Conflict of Interest Statement

The authors declare that the research was conducted in the absence of any commercial or financial relationships that could be construed as a potential conflict of interest.
